# Effect of the JAK/STAT Inhibitor Tofacitinib on Macrophage Cholesterol Metabolism

**DOI:** 10.3390/ijms241612571

**Published:** 2023-08-08

**Authors:** Maria Pia Adorni, Bianca Papotti, Maria Orietta Borghi, Elena Raschi, Francesca Zimetti, Franco Bernini, Pier Luigi Meroni, Nicoletta Ronda

**Affiliations:** 1 Unit of Neurosciences, Department of Medicine and Surgery, University of Parma, Via Volturno 39/F, 43125 Parma, Italy; mariapia.adorni@unipr.it; 2 Department of Food and Drug, University of Parma, Parco Area delle Scienze 27/A, 43124 Parma, Italy; bianca.papotti@unipr.it (B.P.); francesca.zimetti@unipr.it (F.Z.); f.bernini@unipr.it (F.B.); 3 Experimental Laboratory of Immuno-Rheumatologic Researches, IRCCS Istituto Auxologico Italiano, Cusano Milanino, Via Zucchi 18, 20095 Milan, Italy; maria.borghi@unimi.it (M.O.B.); raschi@auxologico.it (E.R.); pierluigi.meroni@unimi.it (P.L.M.)

**Keywords:** tofacitinib, JAK/STAT inhibitor, atherosclerosis, cardiovascular risk, macrophage cholesterol metabolism, cholesterol efflux, cholesterol uptake, cholesterol synthesis

## Abstract

The impact of JAK/STAT inhibitors, which are used in various inflammatory diseases, on cardiovascular risk is controversial and has recently raised safety concerns. Our study investigates the direct effects of tofacitinib on macrophage cholesterol metabolism, which is crucial for atherosclerosis plaque development and stability. Cultured human macrophages THP-1 were used to assess the impact of tofacitinib on cell cholesterol efflux and synthesis via radioisotopic methods, and on cholesterol uptake by measuring the cell cholesterol content with a fluorometric assay. The cholesterol acceptors and donors were either standard lipoproteins or sera from patients with juvenile idiopathic arthritis (JIA) and from control subjects. Tofacitinib significantly increased the macrophage cholesterol efflux to all acceptors; it reduced cholesterol uptake from both the normal and hypercholesterolemic sera; and it reduced cholesterol synthesis. The treatment of macrophages with tofacitinib was able to increase the cholesterol efflux and decrease cholesterol uptake when using sera from untreated JIA patients with active disease as cholesterol acceptors and donors, respectively. In conclusion, our in vitro data support the concept that tofacitinib has a favorable impact on macrophage cholesterol metabolism, even in the presence of sera from rheumatologic patients, and suggest that other mechanisms may be responsible for the cardiovascular risk associated with tofacitinib use in selected patient populations.

## 1. Introduction

Autoimmune and rheumatologic diseases are associated with accelerated atherosclerosis [1,2]. The treatment of these conditions generally reduces cardiovascular risk, but the effects of anti-rheumatic drugs on clinical outcomes and specific atherogenic mechanisms may differ. In particular, considering lipid metabolism, many drugs increase serum lipid levels but induce differing improvements in lipoprotein antiatherogenic function [3,4]. The Janus Kinase/signal transducers and activators of transcription (JAK/STAT) pathway is an essential intracellular signaling mechanism that regulates several cell functions and gene expression, playing a critical role in mediating inflammatory and immune responses [5,6]. Evidence points to a role of the JAK/STAT pathway in atherosclerosis, being its pro-atherogenic induction [7,8] and its atheroprotective inhibition, e.g., through the induction of the ATP binding transporter A1 (ABCA1) expression [9]. Moreover, a blunting effect of the JAK/STAT inhibitor tofacitinib, which is currently indicated in rheumatoid arthritis (RA), psoriatic arthritis, ulcerative colitis, and juvenile idiopathic arthritis (JIA), on oxidized LDL (oxLDL)-induced monocyte adhesion to endothelial cells has been described [10]. Consistently, with such observed favorable effect of tofacitinib, pooled data from six phase III studies and two open-label long-term extension studies on patients with RA demonstrated that tofacitinib use is associated with lower incidences of cardiovascular events [11]. The same research group also reported that a 24-week treatment with tofacitinib in RA patients was associated with increased high-density lipoprotein cholesterol (HDL-C) serum levels, which, in turn, appeared to be associated with a lower risk of major CV events (MACE) [12]. Thus, the JAK/STAT inhibition, in principle, should be a valuable strategy to treat rheumatologic diseases and at the same time reduce cardiovascular risk (CVR). However, unfortunately, other clinical studies produced conflicting results. Tofacitinib use has been associated with an increased risk of MACE in patients over 60 with at least one CV risk factor [13,14,15], with mechanisms not yet clarified. Indeed, the safety concern on JAK/STAT inhibitors has been acknowledged by both American and European drug agencies. Thus, obtaining information on the mechanisms underlying the possible harmful effects of these drugs on the cardiovascular system is urgent. 

As with many anti-rheumatic treatments, tofacitinib use is associated with increased serum lipid levels, but this finding is not “per se” suggestive of increased atherosclerotic risk [16]. In fact, the anti-atherosclerotic properties of circulating lipoproteins usually improve in patients treated with anti-rheumatic treatments [4], including tofacitinib [17,18].

Beyond the serum lipoprotein level and function modifications, tofacitinib may directly modulate macrophage cholesterol handling, an effect that is theoretically very relevant for the understanding of the drug’s reported association with MACE risk. Although the major mechanism identified so far for such effect is that of an increased thrombophilia [19], it is important to confirm or exclude a possible direct effect of tofacitinib on foam cell formation, which is a hallmark of atherosclerosis.

Inflammation and autoimmune reactions enhance monocyte recruiting in vessel intima, macrophage foam cell formation, and cell-free cholesterol accumulation, favoring plaque development and instability and increasing the risk of acute cardiovascular events [20]. Macrophage cholesterol homeostasis is the result of various processes including de novo cholesterol synthesis, cholesterol uptake from circulating lipoproteins, cholesterol esterification via acyl-coenzyme A (CoA):cholesterol acyltransferase (ACAT), reducing the toxic free cholesterol cell content, and cholesterol efflux to extracellular acceptors [21].

Cholesterol uptake from native LDL is mediated by the LDL receptor (LDLr), whose membrane expression is strictly regulated by the intracellular cholesterol content. On the contrary, oxidized LDLs, which are generated in increased amounts during inflammation, are internalized through scavenger receptors, which allow for an uncontrolled cell cholesterol uptake. Cell cholesterol efflux is the first step of reverse cholesterol transport whereby cholesterol is transported to the liver for its elimination [22]. It occurs through aqueous diffusion and membrane receptors such as ABCA1 or ABCG1 and is dependent on the activity of HDLs as extracellular cholesterol acceptors. Immature, lipid-poor HDLs interact preferentially with ABCA1, and more mature lipid-rich particles interact preferentially with ABCG1 [23].

Our study aims to explore the in vitro effect of tofacitinib on macrophage cholesterol homeostasis, namely on the processes of efflux and uptake, using standard lipoproteins as cholesterol donors and acceptors, respectively, and on cholesterol synthesis. In addition, to verify whether tofacitinib also impacts macrophage cholesterol homeostasis in the presence of serum from patients with an inflammatory condition for which the drug is usually indicated, we measured the in vitro cholesterol uptake and efflux in standard macrophages treated with tofacitinib, using the sera of JIA patients who were not treated with the drug, as cholesterol donor and acceptor, respectively.

## 2. Results

### 2.1. Effect of Tofacitinib on Macrophage Cholesterol Efflux

**Figure 1:**
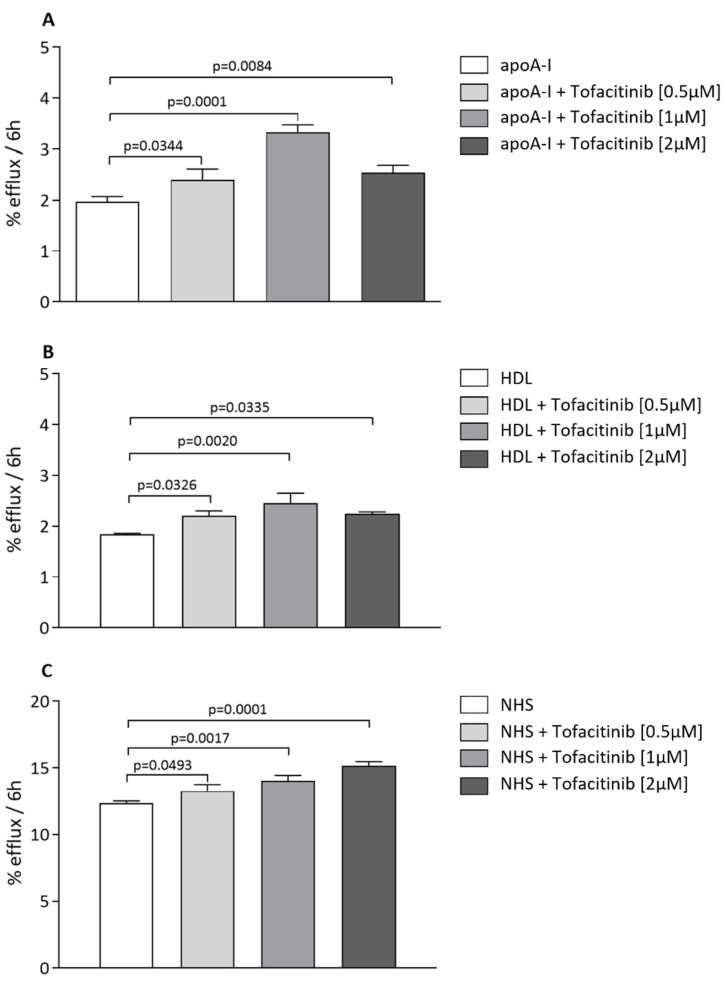
Effect of tofacitinib on cholesterol efflux to various cholesterol acceptors. Cells were radiolabeled with [1,2-^3^H]-cholesterol (2 µC/mL) in the presence of acetylated LDL (acLDL, 25 µg/mL) for 24 h; then, cells were treated without or with tofacitinib at an increasing concentration (0.5, 1, and 2 µM) for 18 h. Cholesterol efflux was promoted to apoA-I (10 µg/mL, panel (**A**)), HDL (12.5 µg/mL, panel (**B**)), and NHS (2% *v*/*v*, panel (**C**)) for 6 h. Cholesterol efflux is expressed as the percent of cholesterol released in the supernatant over total cholesterol incorporated by cells. Statistical significance was calculated via ANOVA with Tukey’s post hoc analysis. Cell treatment was performed in triplicate and data are expressed as means ± SD.

To investigate a possible direct effect of tofacitinib on the macrophage cholesterol efflux, we used a model of human macrophage THP-1 cells loaded with acetylated LDL (acLDL). The cholesterol-loaded THP-1 cells were treated in the presence or absence of tofacitinib at increasing concentrations (0.5, 1, and 2 µM) for 18 h. After this time, cholesterol efflux was promoted to various physiological cholesterol acceptors, namely apolipoprotein A-I (apoA-I) (10 µg/mL), HDL (12.5 µg/mL), and normolipidemic human serum (NHS; 2% *v*/*v*). In acLDL-loaded THP-1 tofacitinib significantly increased cholesterol efflux to apoA-I at all concentrations tested, with a dose-dependent fashion at 0.5 and 1 µM (0.5 µM, *p* = 0.034, +21.8%; 1 µM, *p* = 0.0001, +69%; 2 µM, *p* = 0.0084, +28.9%; Figure 1A). Similarly, tofacitinib significantly increased the macrophage cholesterol efflux to HDL at all concentrations (0.5 µM, *p* = 0.0326, +19.6%; 1 µM, *p* = 0.0020, +33.2%, 2 µM, *p* = 0.0335, +21.7%; Figure 1B), reaching a plateau at 1 µM. Finally, the efflux to NHS was increased by tofacitinib in a dose-dependent manner (0.5 µM, *p* = 0.0493, +7.5%; 1 µM, *p* = 0.0017, +13.8%; 2 µM, *p* = 0.0001, +23%; Figure 1C). In a parallel set of cells, we also evaluated the impact of tofacitinib on cholesterol-efflux-related gene expression via real-time qPCR. In both macrophages with normal cholesterol and acLDL-loaded macrophages, we detected a significant increase in *ABCA1* gene expression after tofacinibib treatment (*p* < 0.01 and *p* < 0.05 in macrophages with normal cholesterol and acLDL-loaded macrophages, respectively) (Supplementary Figure S1A,B), while no significant effect was observed for the *ABCG1* mRNA levels (Supplementary Figure S1C,D).

### 2.2. Effect of Tofacitinib on Macrophage Cholesterol Uptake

**Figure 2:**
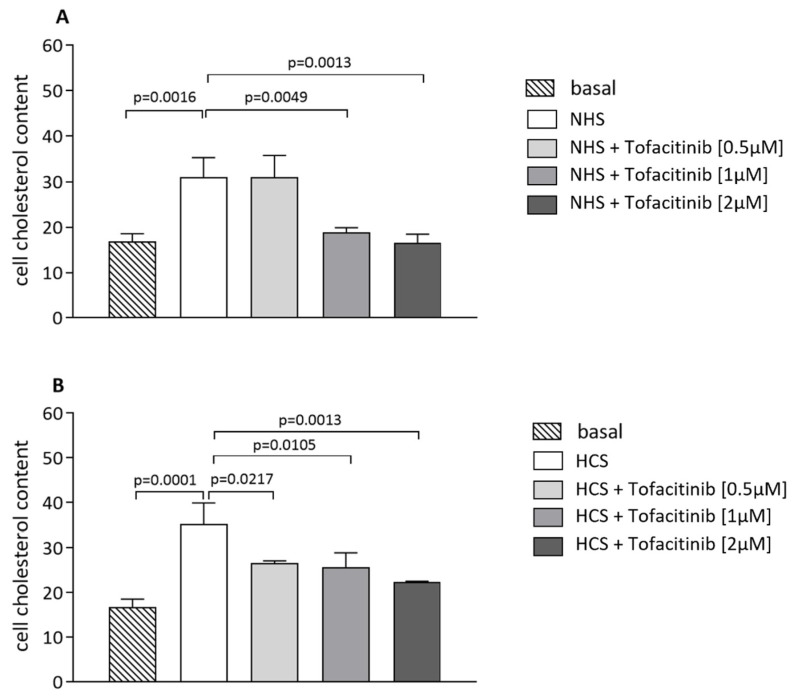
Effect of tofacitinib on cell cholesterol uptake using normal human serum (NHS) or hypercholesterolemic human serum (HCS) as cholesterol donors. Cells were starved with LPDS (5%) for 24 h; then, cells were treated without or with tofacitinib at increasing concentrations (0.5, 1, and 2 µM) for 24 h. Cells were exposed to NHS (10% *v*/*v*) (panel (**A**)) or HCS (10% *v*/*v*) (panel (**B**)) as cholesterol donors. Cell cholesterol content was expressed as µg cholesterol/mg DNA in cell lysates. Statistical significance was calculated via ANOVA with Tukey’s post hoc analysis. Cell treatment was performed in triplicate and data are expressed as means ± SD.

The THP-1 human macrophages were pre-treated with tofacitinib at increasing concentrations (0.5, 1, 2 µM) for 24 h and then exposed to NHS at 10% (*v*/*v*) or to hypercholesterolemic human serum (HCS) for the following 24 h, again in the presence or absence of tofacitinib. Cell exposure to NHS or HCS increased the cell cholesterol content significantly (*p* = 0.0016 and *p* = 0.0001, respectively). This effect was significantly inhibited in the presence of tofacitinib by 39.4% (*p* = 0.0049) and by 48.3% (*p* = 0.0013) at 1 and 2 µM, respectively, using NHS as a cholesterol donor (Figure 2A). In the case of HCS, the cholesterol content increase was inhibited by tofacitinib at all concentrations used, in a dose-dependent manner (0.5 µM, *p* = 0.0217, −24.1%; 1 µM, *p* = 0.0105, −27.1%; 2 µM, *p* = 0.0013, −36.35%) (Figure 2B). When we evaluated the mRNA levels of the *LDLr*, which is one of the main receptors responsible for the cholesterol uptake by cells, we could detect a significant increase induced by tofacitinib in the macrophages both in the basal and cholesterol loading condition (*p* < 0.05 and *p* < 0.01 in macrophages with normal cholesterol and acLDL-loaded macrophages, respectively) (Supplementary Figure S2A,B).

### 2.3. Effect of Tofacitinib on Macrophage Cholesterol Biosynthesis

**Figure 3:**
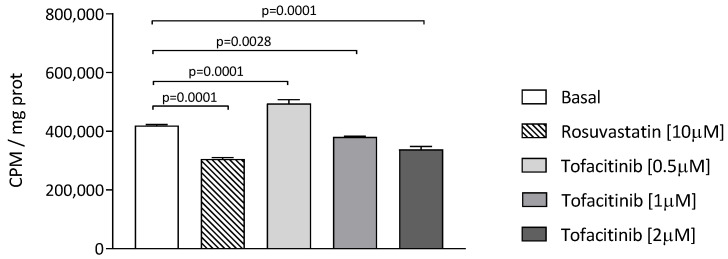
Effect of tofacitinib on macrophage cholesterol biosynthesis. Cells were pre-treated with rosuvastatin (10 µM) or tofacitinib (0.5, 1, and 2 µM) in standard medium or with standard medium alone and then incubated with [2-^14^C]-acetate for 24 h in the presence or absence of the drugs. Cells were lysed in 0.1 M NaOH and total sterols synthesized were purified through thin layer chromatography, and cholesterol radioactivity was measured. Total sterol synthesis was expressed as counts per minute (cpm) per mg of protein. Statistical significance was calculated via ANOVA with Tukey’s post hoc analysis. Cell treatment was performed in triplicate and data are expressed as means ± SD.

The macrophage cholesterol content might also be influenced by cholesterol synthesis. We evaluated the cholesterol biosynthesis in THP-1 human macrophages by measuring the incorporation of radioactive [2-^14^C]-acetate into total cellular sterols in the presence and absence of tofacitinib. The incubation of cells with 10 µM rosuvastatin for 24 h, as expected, reduced the 2-^14^C-acetate incorporation into cholesterol by about 27.2% (Figure 3). Under the same experimental conditions, tofacitinib significantly inhibited cholesterol biosynthesis by 9.1% (*p* = 0.0028) and by 19.4% (*p* = 0.0001) at 1 and 2 µM, respectively. Treatment with tofacitinib at 0.5 µM induced a slight albeit significant increase in cholesterol biosynthesis (+15.3%, *p* = 0.0001) (Figure 3). Finally, we evaluated the influence of tofacitinib on the expression of the gene encoding for the hydroxy-3-methyl-glutaryl-coenzyme A (HMGCoA) reductase, which is the enzyme responsible for the cholesterol biosynthesis, without, however, observing any significant change induced by the drug (Supplementary Figure S3).

### 2.4. Effect of Tofacitinib on Macrophage Cholesterol Efflux to Sera from Healthy Subjects and from Patients with Juvenile Idiopathic Arthritis

**Figure 4:**
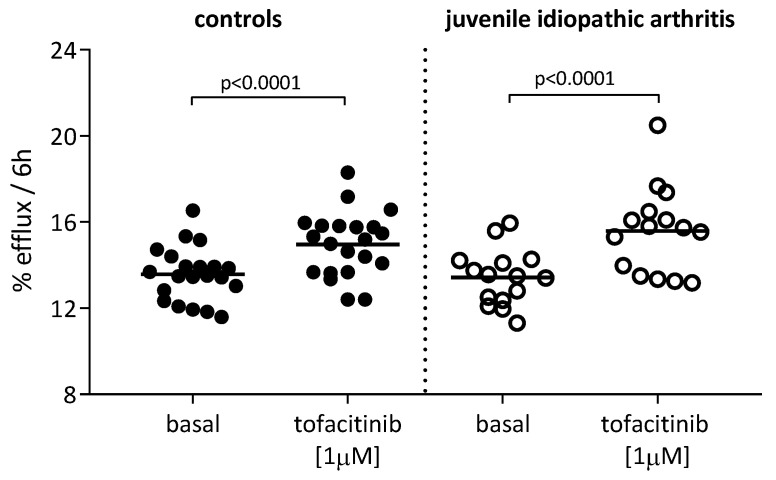
Effect of tofacitinib on cholesterol efflux to control sera and to sera from patients with juvenile idiopathic arthritis (JIA). Cells were radiolabeled with [1,2-^3^H] cholesterol in the presence of acLDL (25 µg/mL) for 24 h and then treated with tofacitinib at 1 µM for 18 h. Cholesterol efflux was promoted to sera (2% *v*/*v*) from control patients (controls, n = 21, filled circles) and from patients with JIA (n = 15, empty circles) for 6 h. Cholesterol efflux was expressed as the percent of cholesterol released in supernatant on total cell cholesterol in cells. Statistical significance was calculated using two-tailed paired Student’s *t*-test.

The AcLDL-loaded THP-1 cells were pre-treated or not treated with tofacitinib 1 µM, which is the most effective concentration for cholesterol homeostasis based on the results described above, before measuring the cholesterol efflux to sera from healthy subjects (controls, n = 21) and from patients with active juvenile idiopathic arthritis (JIA, n = 15), still untreated. Tofacitinib significantly increased the macrophage cholesterol efflux to the controls’ sera by 10.3% (*p* < 0.0001) (Figure 4, left side of the panel) and to the sera from the JIA patients by 16% (*p* < 0.0001) (Figure 4, right side of the panel). 

### 2.5. Effect of Tofacitinib on Macrophage Cholesterol Uptake from Sera from Healthy Subjects and from Patients with Juvenile Idiopathic Arthritis

**Figure 5:**
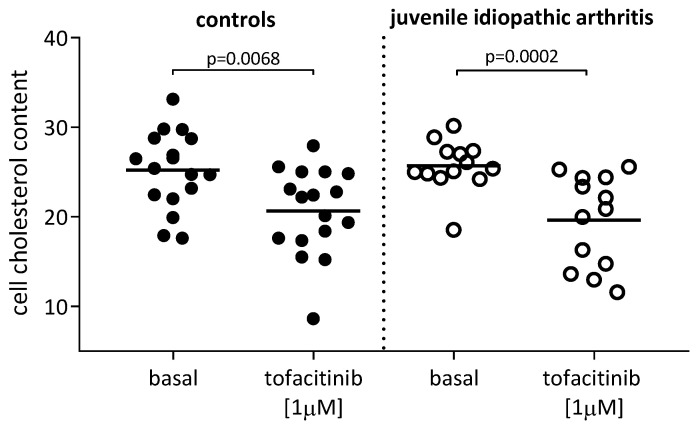
Effect of tofacitinib on cell cholesterol uptake from control sera and from sera of patients with juvenile idiopathic arthritis (JIA). Cells were starved with LPDS (5%) for 24 h; then, cells were treated with tofacitinib at 1 µM for 24 h. Cells were exposed (10% *v*/*v*) to control sera (controls, n = 17, filled circles) or to sera from patients with JIA (n = 13, empty circles). Cell cholesterol content was expressed as µg cholesterol/mg DNA of cell lysates. Statistical significance was calculated using two-tailed paired Student’s *t*-test or Wilcoxon signed-rank test for parameters with normal and skewed distribution, respectively.

As cholesterol uptake is a key step in atherosclerosis development, we tested the effect of tofacitinib on the intracellular cholesterol content in THP-1 human macrophages exposed to whole sera from the same control subjects and from the patients with JIA as above. The treatment of macrophages with tofacitinib significantly decreased the intracellular cholesterol accumulation when using the control sera (*p* = 0.0068, −18%) (Figure 5, left side of the panel) as well as the sera from the JIA patients (*p* = 0.0002; −23.6%) (Figure 5, right side of the panel) as cholesterol donors.

## 3. Discussion

The data on the impact of tofacitinib on cardiovascular disease are still controversial. Indeed, some studies report lower incidences of CV events in RA patients following tofacitinib treatment [11,12], while a recent metanalysis describes an increased risk of MACE with tofacitinib use in subjects with certain CV risk factors and aged older than 65, as compared to anti-TNF treatment [13]. In these cases, the evidence available so far indicates that the major mechanism is increased thrombophilia, but the possible role of tofacitinib in various processes of lipid metabolism has yet to be defined. In this context, tofacitinib might impact the serum lipoprotein levels, serum lipoprotein functions, and vascular macrophage cholesterol handling. The data on serum lipoproteins indicate that tofacitinib induces increased levels that are not necessarily harmful, as the LDL/HDL ratio is unchanged [17]; tofacitinib use is also associated with an increase in the HDL capacity to promote cell cholesterol efflux [18]. In this study, we explored the direct effect of tofacitinib on macrophage functions controlling intracellular cholesterol homeostasis in vitro. We showed that, overall, the tofacitinib effects on human macrophages concur to reduce the intracellular cholesterol content, thus opposing foam cell formation. Our data confirm and add new pieces of information to previous reports obtained with mouse macrophages [24] and with human macrophages treated with proinflammatory stimuli [25].

We explored specific cell cholesterol efflux pathways and showed that tofacitinib increases cholesterol efflux to various extracellular acceptors in cholesterol-loaded macrophages, indicating that this mechanism is not involved in its putative proatherogenic effect. Cholesterol efflux was promoted to ApoA-I, human HDL, and to whole normolipidemic human serum, suggesting the involvement of multiple cholesterol transporters, namely ABCA1, ABCG1, and SR-BI, which display a differential affinity for the above-cited cholesterol acceptors. We further show that in cells treated with tofacitinib, the increased ABCA1-mediated efflux to ApoA-I was associated with a significant induction of *ABCA1* mRNA content. This effect occurred in both macrophages with normal cholesterol and cholesterol-loaded macrophages and suggests a regulatory role of the drug at the transcriptional level. Consistently, it has been previously reported that tofacitinib may reverse the inhibition of ABCA1 expression in macrophages caused by IFNγ, probably through LXRα blocking via STAT [25]. In the same report, the authors describe a similar effect of tofacitinib, although not statistically significant, also for the ABCG1 transporter. The impact of tofacitinib might be dose-dependent and related to the degree of STAT inhibition, with quantitative differences between the various cholesterol efflux pathways, according to our present data and previous evidence [25]. Alternatively, or in addition, tofacitinib might inhibit cholesterol esterification, the process by which cells protect themselves from toxic levels of free cholesterol, thus reducing the cholesterol that is available for efflux. Indeed, it has been reported that JAK-2 stimulates ACAT expression and that such effect can be reversed by its inhibition, leading to increased cell cholesterol efflux [26]. 

Tofacitinib significantly reduced the cholesterol uptake in macrophages in the presence of human serum as a cholesterol donor. Our data seem to exclude this pathway as a possible proatherogenic mechanism. This is true not only when using normolipidemic human serum as a donor, but also in the presence of hypercholesterolemic serum from familial hypercholesterolemic (FH) patients. In the first case, the receptor involved is mainly the LDLr, which interacts with native LDL and is regulated by intracellular cholesterol concentration; in the second case, mainly scavenger receptors are involved, interacting with modified LDL, which is typically present in hypercholesterolemic sera, in an uncontrolled manner. Our findings on cholesterol uptake are consistent with the reported role of the JAK-STAT pathway in the expression of scavenger receptors [27]. Furthermore, the observed increasing effect of tofacitinib on the levels of *LDLr* mRNA is not surprising because of the feedback response to intracellular cholesterol reduction involving the sterol-regulatory element (SRE)-1 in the LDL receptor promoter [28,29] and a family of SRE-binding proteins (SREBPs), namely SREBP1 and SREBP2 [30,31].

Finally, our in vitro study showed that macrophage cholesterol synthesis responded differently to tofacitinib concentrations, namely with an increase for 0.5 µM and with a decrease for 1 and 2 µM. We observed a discrepancy between the tofacitinib impact on the HMGCoA reductase function and gene expression; the former is significantly inhibited, whereas the latter does not seem to be affected. This observation suggests a post-transcriptional regulation by tofacitinib that still needs to be elucidated.

By combining the results of the uptake and synthesis experiments, it seems that at 0.5 µM, tofacitinib does not impact the intracellular cholesterol content due to a balance between the reduced uptake and increased synthesis, while at higher concentrations, macrophage cholesterol depletion occurs due to both uptake and synthesis reduction. Overall, the tofacitinib concentration that better reduces the macrophage cholesterol content in vitro is 1 µM, corresponding to the circulating levels in the patients treated with the drug [32]. It is impossible to draw conclusions on the actual in vivo effects of tofacitinib serum concentrations on the macrophage cholesterol content in treated patients; also, the generation of several hepatic metabolites of tofacitinib might contribute to its activity in vivo [33]. However, our observation of a dose-dependent effect of tofacitinib on all pathways concurring to intracellular cholesterol homeostasis suggests that tofacitinib concentration is a critical factor for atherogenesis or atheroprotection with respect to plaque development.

Considering that tofacitinib is indicated in inflammatory conditions, we explored its effect on macrophage cholesterol homeostasis in the presence of serum from JIA patients with active disease to verify whether the studied drug activity was the same. When treating macrophages with 1 µM tofacitinib, the concentration mostly effective in reducing the intracellular cholesterol, we showed that the cell cholesterol efflux was equally increased when using sera from patients with JIA or from healthy controls as acceptors. As sera from autoimmune inflammatory diseases might contain inflammatory molecules and dysfunctional lipoproteins possibly affecting cell cholesterol efflux [23,34,35], our observation indicates that 1 μM tofacitinib is able to promote this process even in potentially unfavorable conditions. Similarly, we observed a significantly reduced cholesterol uptake in macrophages upon treatment with 1 μM tofacitinib when using sera from patients with JIA as cholesterol donors, similar to that observed with sera from healthy subjects. The differences in age, gender distribution, and lipid profile between the two groups might be a limitation; however, our aim was not to compare the absolute values of the parameters relative to lipoprotein function between the groups, but rather to verify whether direct tofacitinib activity on macrophage cholesterol metabolism could also function in the presence of an inflammatory milieu, which is a hypothesis that was confirmed.

Our findings are in line with clinical studies indicating that tofacitinib treatment is associated with a block of carotid atherosclerosis progression [36] and with a regression of atherosclerosis in animal models [24], suggesting an inhibitory effect on foam cell formation.

In conclusion, our in vitro data support the concept that tofacitinib has a favorable impact on macrophage cholesterol metabolism, even in the presence of serum from rheumatologic patients, and suggest that other mechanisms may be responsible for the cardiovascular risk associated with tofacitinib use in selected patient populations.

## 4. Materials and Methods

Cells. Human monocyte-derived macrophages THP-1 were grown in cell culture medium RPMI1640 (Euroclone, Milan, Italy) with 10% *v*/*v* Fetal Calf Serum (FCS; Euroclone, Milan, Italy) in the presence of 0.5% *v*/*v* gentamicin and 2.5 g/L *w*/*v* glucose (both from Merck, Darmstadt, Germany), and 1 mM sodium pyruvate and 0.05 mM 2-mercaptoethanol (both from Thermo Fisher Scientific, Waltham, MA, USA). To allow for THP-1 differentiation into macrophages, cells were cultured in 24- or 12-well plates, based on the specific assay requirement, in the presence of 100 ng/mL phorbol 12-myristate 13-acetate (PMA; Merck, Darmstadt, Germany) for 72 h.

Cholesterol efflux. Fully differentiated human THP-1 macrophages were radiolabeled for 24 h with 2 µCi/mL of 1,2-^3^H-cholesterol (Perkin Elmer, Waltham, MA, USA) in the presence or absence of 25 µg/mL of acetylated-LDL and 2 µg/mL acetyl-coenzyme A:cholesterol acyltransferase (ACAT) inhibitor (Merck, Darmstadt, Germany) to maintain cholesterol in its unesterified form. Then, cells were treated for 18 h with a solution of RPMI 1640 supplemented with 0.2% *w*/*v* Bovine Serum Albumin (BSA) and tofacitinib (both from Merck, Darmstadt, Germany) at the concentrations of 0.5, 1, and 2 µM (concentrations corresponding to the mean circulating levels of the molecule during therapy) [37]. Finally, cholesterol efflux was promoted for 6 h to either 10 µg/mL apolipoprotein A-I (apoA-I), 12.5 µg/mL native HDL (both from Merck, Darmstadt, Germany), or 2% *v*/*v* of normal human serum obtained from a pool of normolipidemic subjects. In another set of cells, using the above-described experimental conditions, cholesterol efflux was promoted to 2% *v*/*v* sera obtained from either patients with juvenile idiopathic arthritis (JIA) (n = 15) or healthy controls (n = 21). To check for adequate cell responsiveness, apoA-I and a standard serum obtained from a pool of normolipidemic subjects were tested together with serum samples in each assay. The amount of radioactive 1,2-^3^H-cholesterol in each sample was determined using the Liquid Scintillation Counter TRI-CARB 4810TR (Perkin Elmer, Whaltham, MA, USA). The relative cholesterol efflux values were used to normalize the different experiments to correct for the inter-assay variability. Cholesterol efflux, expressed as the percentage of 1,2-^3^H-cholesterol released in the culture medium over the total intracellular radioactivity, was the mean of three independent experiments. 

Cholesterol uptake. Fully differentiated human THP-1 macrophages were treated with RPMI 1640 supplemented with 0.5, 1, and 2 µM tofacitinib for 24 h. Subsequently, macrophages were incubated with 10% *v*/*v* of human serum obtained either from a pool of normolipidemic subjects (NHS; normal human serum) or from familial hypercholesterolemia (FH) patients (HCS; hypercholesterolemic serum) for an additional 24 h. In another set of experiments, we used as cholesterol donor 10% *v*/*v* sera from either patients with juvenile idiopathic arthritis (JIA; n = 13) or from healthy subjects (n = 17). All subjects gave written informed consent, and the experimental procedures followed the Declaration of Helsinki. At the end of the incubation with sera, cell monolayers were lysed in 1% sodium cholate solution (Merck, Germany) supplemented with 10 U/mL DNase (Merck, Germany). Cholesterol was then measured fluorometrically on a Tecan Spark 10 M (Tecan, Männedorf, Switzerland) microplate reader, using the Amplex Red^®^ Cholesterol Assay Kit (Thermo Fisher Scientific, Waltham, MA, USA) and following the manufacturer’s instructions [38]. An aliquot of cell lysates was used to measure DNA content via Diphenylamine reagent [39]. To check for adequate cell responsiveness, sera obtained from pools of normolipidemic and hypercholesterolemic subjects were tested together with serum samples in each assay. These values were used to normalize the different experiments in order to correct for inter-assay variability. Macrophage cholesterol content, expressed as total micrograms of intracellular cholesterol after the incubation with sera per milligram of DNA, was the mean of three independent experiments.

Cholesterol biosynthesis. The synthesis of cholesterol was determined by measuring the incorporation of radioactive [2-^14^C]-acetate into total cellular sterols [40]. Fully differentiated human THP-1 macrophages were pre-treated with 10 µM rosuvastatin or 0.5, 1, or 2 µM tofacitinib for 24 h. Cells were then incubated for an additional 24 h with 2 μCi/mL of [2-^14^C] acetate (Perkin Elmer, Waltham, MA, USA) in the presence or absence of drugs at the concentrations previously described. Cell monolayers were washed with phosphate-buffered saline (PBS; Euroclone, Milan, Italy) and digested with 0.1 M NaOH overnight at 4 °C under gentle shaking. Each sample was saponified at 60 °C for 1 h in alcoholic KOH solution after the addition of 10^5^ cpm/sample of 1,2-^3^H-cholesterol (Perkin Elmer, Waltham, MA, USA) as an internal standard. The unsaponifiable material was extracted with petrol ether and counted for radioactivity. To evaluate the incorporation of labelled acetate into cellular sterols, these were separated from the unsaponifiable fraction via thin-layer chromatography by using low-boiling point petroleum ether/diethyl ether/acetic acid (70:30:1). Radioactivity was measured via liquid scintillation counting. In parallel, BCA Assay (Thermo Fisher Scientific, Waltham, MA, USA) was used to determine the total protein content in each sample; cholesterol synthesis was expressed as total 2-^14^C cpm over milligrams of proteins and was the mean of three independent experiments. 

Gene expression analyses. See Supplementary Methods.

Study participants. Patients with JIA were diagnosed according to the European guidelines based on laboratory and radiological findings, and based on clinical examination [41] and the experimental protocol was approved by the Ethics Committee of the Istituto Auxiologico Italiano (protocol code 2017_12_19_02, 19 December 2017). Healthy controls were chosen from the DIALER database [42] for which the approval of the Ethical Committee of the University of Bologna was previously obtained. The study was conducted in accordance with the Declaration of Helsinki and patients gave written informed consent.

Demographic and lipid profiles of control healthy subjects (controls) and patients in the active phase of juvenile idiopathic arthritis (JIA) are presented in Table 1. JIA patients were significantly younger than control subjects (*p* = 0.0023). Similarly, gender distribution was slightly albeit significantly different between the two groups (*p* = 0.0025), with the highest percentage of women within JIA patients. Total cholesterol, HDL-C, and triglyceride concentrations were similar between the two populations (all *p* > 0.05). However, JIA patients displayed slightly lower LDL-C concentrations compared to controls (*p* = 0.0035).

## Figures and Tables

**Table 1:** Age, gender, and lipid profiles of JIA patients and controls. HDL-C: high-density lipoprotein cholesterol; LDL-C: low-density lipoprotein cholesterol; ns: not significant. Normally distributed continuous parameters are presented as mean ± SD and skewed continuous parameters are expressed as median (interquartile range defined as 25th percentile, 75th percentile). Significant differences were calculated using two-tailed paired Student’s *t*-test or Wilcoxon signed-rank test for parameters with normal and skewed distribution, respectively. A *p* value < 0.05 was considered statistically significant and indicated in bold.

	Controls (n = 21)	JIA (n = 15)	*p*-Value
**Demographic parameters**			
Age	49 (30, 65)	26 (22, 31)	***p* = 0.0023**
Gender (%)	F = 68.2	F = 85.7	***p* = 0.0025**
**Biochemical parameters**			
Total cholesterol (mg/dL)	222.5 (186.8, 235.3)	190 (169.3, 222.3)	ns
HDL-C (mg/dL)	56.5 (51.5, 69.0)	67 (54.25–84.5)	ns
LDL-C (mg/dL)	136.00 ± 29.95	110.60 ± 31.36	***p* = 0.0159**
Triglycerides (mg/dL)	76.5 (59.8, 127.5)	90.5 (63.5, 97.8)	ns

## Data Availability

The authors declare that the data generated and analyzed during this study are included within the article and Supplementary Materials.

## Supplementary Materials

The following supporting information can be downloaded at https://www.mdpi.com/article/10.3390/ijms241612571/s1.
