# Supplementary material for: Effect of the JAK/STAT Inhibitor Tofacitinib on Macrophage Cholesterol Metabolism
Source: Int J Mol Sci. 2023 Aug 8;24(16):12571. doi: 10.3390/ijms241612571 (PMC10454555; doi:10.3390/ijms241612571)
Supplement: Supplementary file 1 [file ijms-24-12571-s001.zip › ijms-2556146-supplementary.pdf]

SUPPLEMENTARY MATERIALS

Article

Effect of the JAK/STAT inhibitor tofacitinib on macrophage cholesterol metabolism

Maria Pia Adorni[1], Bianca Papotti[2], Maria Orietta Borghi[3], Elena Raschi[3], Francesca Zimetti[2], Franco Bernini[2], Pier Luigi Meroni[3], Nicoletta Ronda [2,*]

SUPPLEMENTARY METHODS

Gene expression analyses. Total RNA was extracted from cell monolayers through the TRIzol™ Reagent (ThermoFisher Scientific, Waltham, MA, USA) following the manufacturer's instructions. RNA concentration and purity was determined using a NanoDrop™ 2000 spectrophotometer (ThermoFisher Scientific, Waltham, MA, USA), observing a mean concentration of 229 ng/µl and a purity of 1.8 (absorbance ratio at 260 and 280 nm). cDNAs were then retro-transcribed starting from 1µg RNA for each sample using the qScript™ cDNA superMix commercial kit (Quanta Biosciences, Beverly, MA, USA). Gene expression analyses were measured with TaqMan® assays (Applied Biosystems, Waltham, MA, United States), as follows: *ABCA1* (Hs01059101_m1), *ABCG1* (Hs00245154_m1), *LDLR* (Hs0192524_m1), and *HMGCR* (Hs00168352_m1) on a QuantStudio™ 3 Real-Time PCR instrument (ThermoFisher Scientific, Waltham, MA, USA). The ΔΔCt method was applied to determine the mRNA levels in each reaction, using the β2-Microglobulin (Hs99999907_m1) as the normalizer internal gene, and expressed as relative quantification (RQ) to a the basal condition.

SUPPLEMENTARY FIGURES

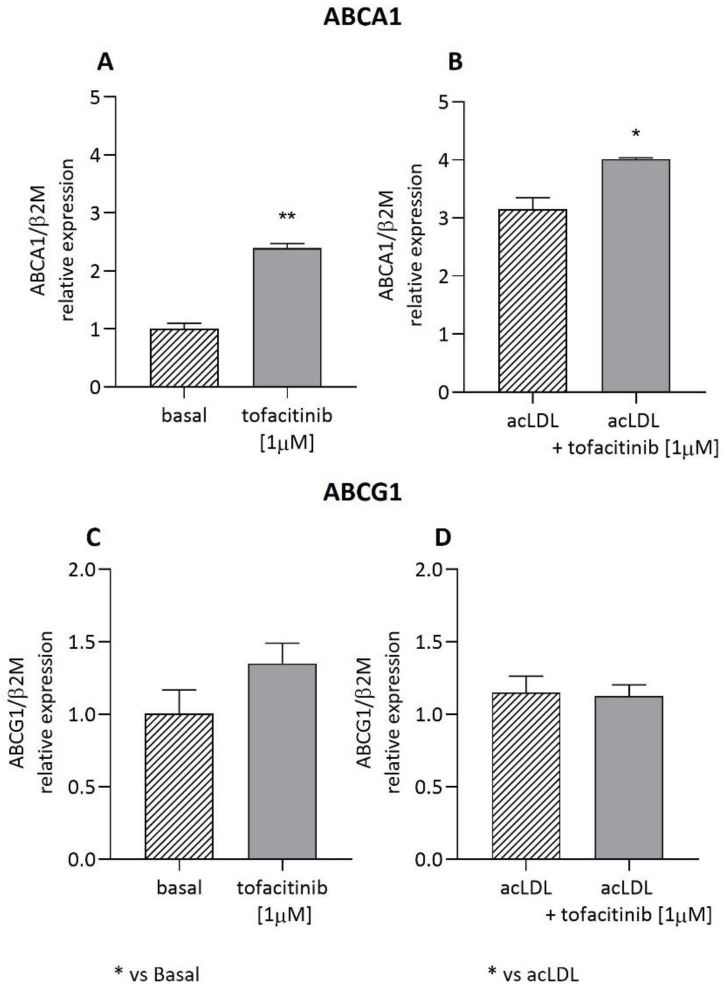

Supplementary Figure S1. Effect of tofacitinib on *ABCA1* and *ABCG1* gene expression. Cells in basal conditions (A and C) or previously incubated with acetylated LDL (acLDL, 25 μg/ml) for 24 h (B and D) were treated with tofacitinib [1 μM] for 18h. At the end of the incubation total RNA was extracted and real time qPCR was performed. Experiments were performed in duplicate and data expressed as mean ± SD. Statistical analyses were performed using the unpaired t-test * $p < 0.05$; ** $p<0.01$

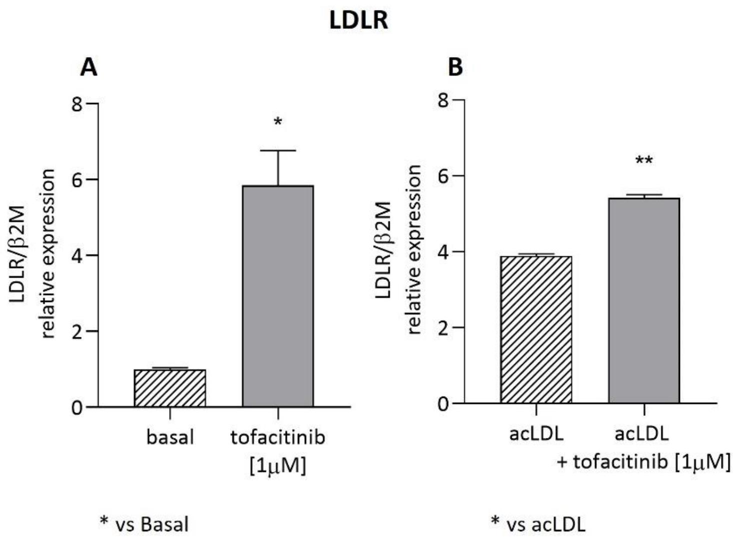

Supplementary Figure S2. Effect of tofacitinib on *LDLR* gene expression. Cells in basal conditions (A) or incubated with acetylated LDL (acLDL, 25 μg/ml) for 24 h (B) were treated with tofacitinib [1 μM] for 18h. At the end of the incubation total RNA was extracted and real time qPCR was performed. Experiments were performed in duplicate and data expressed as mean ± SD. Statistical analyses were performed using the unpaired t-test * $p < 0.05$; ** $p<0.01$

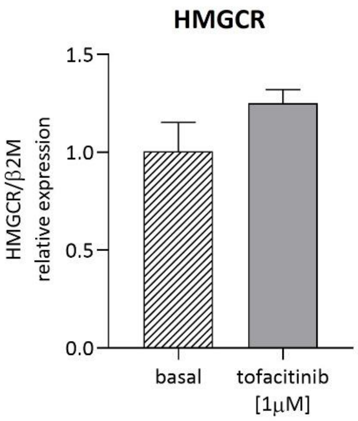

Supplementary Figure S3. Effect of tofacitinib on *HMGCR* gene expression. Cells were incubated with tofacitinib [1 μM] for 18h. At the end of the incubation total RNA was extracted and real time qPCR was performed. Experiments were performed in duplicate and data expressed as mean ± SD. Statistical analyses were performed using the unpaired t-test.
